# Engaging Older Adults in Climate Action: Insights Into Attitudes, Motivations, and Contributions

**DOI:** 10.1093/geroni/igaf122.1263

**Published:** 2025-12-31

**Authors:** Paola Zaninotto, Karl Pillemer

## Abstract

Older adults are among the most vulnerable to the effects of climate change, yet their perspectives, behaviours, and potential contributions to climate action remain underexplored. This symposium brings together new research on attitudes, motivations, and engagement with climate change among older adults, offering insights into how this growing demographic perceives climate risks and contributes to environmental sustainability. The session will open with findings from the English Longitudinal Study of Ageing (ELSA), highlighting distinct attitudes towards climate change risk among older adults in England and their demographic, social, and economic correlates. Next, we explore eco-generativity, how exposure to nature fosters both social and environmental responsibility, and its implications for promoting pro-environmental behaviours across the lifespan. We then turn to older climate activists, examining the key motivational pathways driving their engagement and the role of social connections, emotional ties to nature, and lifelong learning in sustaining their activism. A comparative study of older adults in Italy and Sweden further contextualises these themes, shedding light on the interplay between environmental attitudes, generational responsibilities, and activism. Finally, we present a new set of validated measures designed to assess climate knowledge, concerns, and actions among older adults, providing tools to better integrate climate change into ageing research and policy. By weaving together these diverse yet complementary perspectives, this symposium advances our understanding of older adults as active agents in climate resilience and sustainability, challenging assumptions of disengagement and highlighting strategies to enhance their involvement in environmental action. Climate and Aging Interest Group Sponsored Symposium

